# Searching for the elusive typhoid diagnostic

**DOI:** 10.1186/1471-2334-10-45

**Published:** 2010-03-05

**Authors:** Stephen Baker, Michael Favorov, Gordon Dougan

**Affiliations:** 1 Oxford University Clinical Research Unit, The Hospital for Tropical Diseases, Ho Chi Minh City, Vietnam; 2 The Centre for Tropical Medicine, Nuffield Department of Clinical Medicine, Oxford University, Oxford, UK; 3 The International Vaccine Institute, Kwanak-gu, Seoul, Korea; 4 The Wellcome Trust Sanger Institute, Hinxton, Cambridge, UK

## Abstract

Typhoid (enteric) fever is still a common disease in many developing countries but current diagnostic tests are inadequate. Studies on pathogenesis and genomics have provided new insight into the organisms that cause enteric fever. Better understanding of the microorganisms explains, in part, why our current typhoid methodologies are limited in their diagnostic information and why developing new strategies may be a considerable challenge. Here we discuss the current position of typhoid diagnostics, highlight the need for technological improvements and suggest potential ways of advancing this area.

## Background

Enteric (typhoid) fever remains a common disease in many parts of the world where access to clean water is limited. In places such as India, Nepal, Pakistan, Indonesia and parts of sub-Saharan Africa typhoid is still a substantial public health problem [1-4]. In these areas, febrile disease is common, so accurate diagnosis facilitates treatment selection, particularly as antimicrobial resistance is emerging [5]. Enteric fever is an all encompassing term for the disease caused by several serovars of *Salmonella enterica *including (*S*.) Typhi and (*S*.)Paratyphi A. Although globally *S*. Typhi is the most common cause, *S*. Paratyphi A infections occur in significant numbers in some parts of the world and is often associated with travelers [6-9]. In contrast, *S*. Paratyphi B and C are relatively uncommon. This article focuses specifically on *S*. Typhi and the disease it causes; typhoid. With respect to other invasive *Salmonella, S*. Typhi causes a greater disease burden and there is a superior level of understanding of this organism. However, all of the arguments presented here are poignant for the diseases caused by other human invasive *Salmonella *pathovars.

Despite WHO recommendations, few countries have taken on typhoid immunization [10], this is in part related to uncertainties about disease burden. The best incidence assessment is based on available, sparse surveillance information, estimated that in 2000 there were 21,650,974 illnesses and 216,510 deaths due to typhoid and that paratyphoid caused 5,412,744 illnesses [2]. These data is extrapolated from limited studies and such figures, therefore, may be imprecise, this is compounded by a lack of accurate diagnosis. Therefore, new diagnostics will play a key role in decreasing the incidence of typhoid fever, by permitting governments to accurately assess the particular burden of disease and apply vaccination regimes accordingly. The development of cheap and reliable enteric fever diagnostics would undoubtedly benefit long term disease control and treatment.

Presently, direct blood culture, followed by microbiological identification is the gold standard, any potential new test needs to offer a higher diagnostic rate than this procedure [11]. Blood culturing of *S*. Typhi, whilst considered "routine", is expensive and requires specialist facilities and personnel. Furthermore, *S*. Typhi and *S*. Paratyphi A are not always culturable even if good microbiological facilities are available. Diagnostics based on serology, antigen detection or DNA are available but have limitations. In the document entitled 'The diagnosis, treatment and prevention of typhoid', the WHO state that 'the method used as the gold standard for the laboratory diagnosis of typhoid should approach 100% each for sensitivity, specificity, and positive and negative predictive values'[11]. However, current tests need significant improvement to reach such rigorous standards.

**Figure 1:**
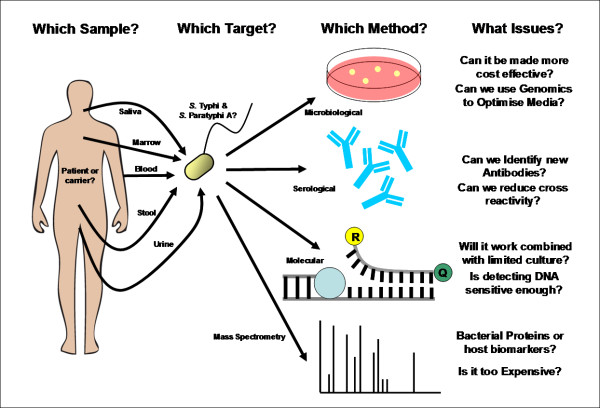
**Identifying the techniques and issues which surround the development of a new enteric fever diagnostic test. Samples, targets, methods and issues**.

In view of these problems, is the goal set by WHO achievable and what are the barriers? Significant advances have been made in our understanding of the biology and genomics of both *S*. Typhi and *S*. Paratyphi A [12-15]. Using this information we can reassess typhoid diagnostics and consider the potential and the limitations of different approaches (Figure 1).

## Typhoid infections

**Figure 2:**
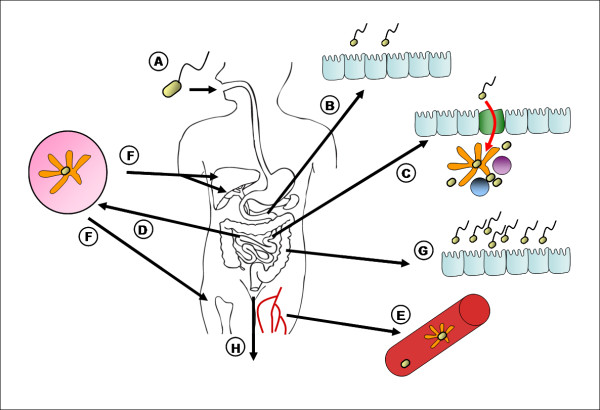
**The lifestyle of *Salmonella *Typhi in the human host and implications for diagnostics**. A; For *S*. Typhi infection, the organism normally enters the human host through oral ingestion of an infectious dose. B; *S*. Typhi does not replicate in large numbers in the intestine and shedding may be sporadic and limited. C; Invasion occurs through the terminal ileum, perhaps a short time after ingestion, M cells may be the preferred portal of entry. D; *S*. Typhi is transferred to monocytic cells and is trafficked to the reticulo-endothelial system, potentially in a semi-dormant state. E; *S*. Typhi re-emerges at an unknown time from the reticulo-endothelial system, possibly as the acquired immune response is activated, and re-enters the blood stream in low numbers. F; *S*. Typhi seeds into the liver, the gall bladder and the bone marrow where it can reside and may be detected for months or years. G; *S*. Typhi can enter into the bile duct and be shed sporadically, potentially in high numbers into the environment via the intestine.

*S*. Typhi causes typhoid, a fecal-oral infection leading to systemic disease (Figure 2). Tissue invasion drives a potentially close encounter with the immune system. However, *S*. Typhi is an immuno-modulatory pathogen which goes to great lengths to avoid detection by the immune host defenses. The pathogenesis of typhoid fever in man has received only limited attention. This is mainly because both *S*. Typhi and *S*. Paratyphi A are host-restricted to humans and there is no known zoonotic reservoir. Experimentation using surrogate hosts and *S. enterica *serotypes (e.g. *S*. Typhimurium) suggests that tissue invasion occurs predominantly through M cells on Peyer's patches in the terminal ileum (Figure 2) [16,17]. Whilst these mechanisms have never been directly proven for typhoid, it is clear that *S*. Typhi has predominantly forsaken ongoing transmission in the habitat of the mammalian gastrointestinal tract of most enteric bacteria, in the favor of systemic dissemination. The infection eventually localizes to the bone marrow and ultimately the gall bladder where the internal transmission cycle is completed as organisms are shed in bile, potentially in high numbers (Figure 2).

*S*. Typhi (and *S*. Paratyphi A) is highly clonal, exhibiting limited genome variation, suggesting this organism is recently evolved [15,18]. The genetic basis of the switch from an enteric to a systemic lifestyle is imprinted in its genetic makeup. Many genes associated with intestinal persistence (e.g. *shdA, ratB*) or interaction with host surfaces (e.g. fimbria, pili etc.) are inactivated, limiting potential mechanisms for colonizing within and between hosts. For example, genes that contribute to fluid release (e.g. *sopA*) or intracellular survival (e.g. *sopE2, sseJ*,) are inactivated [13,18]. Thus, invading *S*. Typhi may follow a simple default pathway favoring limiting activation of the inflammatory response. *S*. Typhi (but not *S*. Paratyphi A) also expresses the Vi capsular polysaccharide, that possesses immuno-modulatory properties, potentially further dampening the immune response [19,20]. One of the consequences of minimal early inflammation is a lack of the classical gastroenteritis associated with other gastrointestinal pathogens. Additionally, humans do not react clinically to the initial invasion step and there is an incubation period before disease symptoms emerge, which occurs during the systemic phase of infection. This stage is one of the early confounders to typhoid diagnostics, the temporal distance between infection and disease hinders the detection of the organism.

The presence of bacteria in any tissue may be transient, as cells traffic or become activated. Thus, trafficking in blood may only occur during a limited window, making a positive blood culture challenging. This situation may be exacerbated as many patients reach microbiological facilities at a later stage of infection or may be "self treated" with antimicrobials. Thus, there are a number of characteristics of the pathogenesis of typhoid that impinge on diagnostics [21,22]. An obvious caveat is the low number of bacteria in the blood and other tissues. It is theoretically possible that there are actually high levels of organisms in the blood but that these are present in an 'unculturable' form. For example, the rapid transfer of bacteria in a semi-quiescent form from within an intracellular vacuole to laboratory media may kill this fastidious and delicate organism. *S*. Typhi is certainly less robust than many other *Salmonella*. Bone marrow is the most sensitive culture route but this is an invasive procedure and is seldom performed outside specialist hospitals. Volunteers challenged with virulent and attenuated *S*. Typhi strains only shed the organism sporadically in stools, potentially compromising the stool culturing approach [23,24].

Many systemic pathogens exhibit the ability to undergo antigenic variation, thus allowing the organism to divert the immune response. Analysis of multiple *S*. Typhi genomes shows a lack of obvious evidence for any amount of immune selection on the organism. Furthermore, no known *S*. Typhi antigens exhibit significant evidence for variation, a fact highlighted by comparing gene sequences between phylogenetically representative *S*. Typhi [12]. These data provide further evidence of the ability of *S*. Typhi to cause a systemic infection without stimulating a significant inflammatory response and transfer from the gastrointestinal lumen to the reticuloendothelial system in a relatively undetected fashion. Indeed, one may argue that the ability of *S*. Typhi to avoid immune detection constitutes the organism as a "stealth" pathogen and this has significant implications for diagnostics.

The lack of immune selection on the organism suggests that *S*. Typhi predominantly occupies an privileged niche within the host, a predominantly intracellular pathogen that can survive for long periods in this state. Indeed, frequent relapses associated with the recrudescence of the pathogen and the lack of immune protection in typhoid patients to re-infection further supports a dampening of immunity [25]. Thus, *S*. Typhi may induce only weak immunity, including a muted antibody response. It is worth noting that in typhoid endemic areas many individuals who have never reported typhoid exhibit serological evidence that they have been infected by the pathogen [26]. Thus, it is highly feasible that many people who get exposed by *S*. Typhi do not progress to develop the recognized disease syndrome, or individuals have a small amount of constant boosting due to prolonged exposure.

An additional caveat for an effective and appropriate diagnostic test is the cross section of organisms that can cause a disease syndrome that is, clinically, indistinguishable from typhoid fever. Depending on the location, a number of viral, parasitic and bacterial pathogens can mimic the basic features of typhoid thus confounding the issues of sample collection, clinical management and efficient diagnosis.

## The limitations of microbiological culture

*S*. Typhi are ordinarily cultured from 5-10 ml of blood in 30-50 ml of broth. The probability of recovering organisms is increased at greater blood volumes, compromising diagnosis in children. In the developed world, blood culture is semi-automated, exploiting sophisticated culture apparatus. Blood taken from patients is inoculated into vessels which are designed to fit in specific machines and contain specialized media, often there is minimal or no dilution of the sample into this media. In this way, the whole process can be captured by an integrated system and a particular laboratory may become dependent on the same supplier, which has particular financial constraints. The advantage of this approach is that it improves specificity and standardization.

The main limitation to the wide spread distribution of semi-automated blood culture systems is cost. Blood culture facilities are rare in many developing countries, often limited only to major hospitals in large cities. Access to receiving a blood culture becomes, therefore, the limiting factor to typhoid diagnosis. It is worth speculating that alternative culture systems, made available at a lower cost and less dependent on expensive consumables, could encourage more facilities to be established in poorer regions. It is also worth noting that in a single tropical setting blood infections may be caused by a wide range of other gram negative and gram positive organisms (e.g. *S*. Typhi, Streptococci, Leptospira, etc.), parasites (e.g. Plasmodium) and viruses (e.g. dengue) [27,28]. Blood culture may or may not be a suitable assay for a specific infection, depending on the pathogen and the location.

Taken that there are only low levels of *S*. Typhi present in blood, how might we improve approaches to direct culture? Could *S*. Typhi culture be further optimized, by taking advantage of some atypical biochemical properties of the organism? Examination of the *S*. Typhi genome highlights metabolic and scavenging pathways inactivated by the accumulation of pseudogenes [18]. Examples include the cobalamin pathway, many metabolic transporters and iron uptake systems. Understanding specific biochemical pathways that are up-regulated under defined conditions may permit some modeling of conditions in which *S*. Typhi can be grown more efficiently. In short, could we use a method that we define as "metabolomic modeling" to design recovery media to enrich for *S*. Typhi? This approach is certainly worth considering but may only have a marginal effect on bacterial recovery.

Ultimately, the low level of bacteria in the sample may set an impenetrable practical barrier which may only be circumvented by purification or enrichment technology. The culturing of bone marrow biopsies is more sensitive than that of blood culture and a modified technique to take bone marrow in a more straightforward and somewhat less brutal manner would be desirable [29].

## The limitations of serology

The first typhoid diagnostic, the Widal test, was developed in 1896. The methodology is dependent on agglutination; *S*. Typhi cells are used to detect antibodies in blood. This crude assay is a visual test that monitors agglutinating antibodies that react with *S*. Typhi [30]. Problems associated with the use of Widal are somewhat obvious and may apply to other serologically based assays for typhoid. *S*. Typhi is a relatively invariant pathogen so antigenic variation *per se *should not be a significant confounder. However, *S*. Typhi is a member of the *Enterobacteriaceae*. Many of the surface antigens of the *Enterobacteriaceae *demonstrate significant conservation and induce antibodies that are cross-reactive. Consequently, as humans mature they accumulate antibodies that are cross-reactive with *S*. Typhi. Thus, it may be impossible to develop a specific diagnostic kit for typhoid using semi-purified antigens. Indeed, any such kit would likely yield significant false positives.

*S*. Typhi expresses a number of immunogenic structures on the surface, some of which provide a basis for serology identification. These include O (lipopolysaccharide), H (flagella) and the somewhat less immunogenic Vi capsule. *S*. Typhi exhibiting variation in these antigens are uncommon, with notable exceptions. *S*. Typhi found in Indonesia express variant H antigens including H:j and H:z66 [31-34]. Vi-negative *S*. Typhi isolates have been reported in Pakistan but are rare [35,36]. Therefore, *S*. Typhi expressing O (O9, O12), Vi and H:d are ubiquitous in most endemic areas. Sero-prevalence studies have been performed in endemic regions to determine antibody titers to O, H and Vi in the general population [26,37]. Many individuals in endemic areas have cross-reactive antibodies even though they have no clinical record of typhoid. Additionally, such raised antibody levels frequently cannot be detected in patients with culture confirmed typhoid. Problems have also been encountered during the testing of commercial serological tests, including Typhidot and Tubex [38,39]. These assays were assessed in population-based typhoid surveillance studies in several countries and in all locations the sensitivity and specificity for Tubex and Typhidot was only around 70% and 80% respectively [40,41].

Clearly the abundance and avidity of anti-*S*. Typhi antibodies varies and it is difficult to imagine how a clean diagnostic assay with high specificity could be produced targeting these classical antigens. Can other antigen/antibody complexes be used as more accurate diagnostics? This is an under-studied area with few *S*. Typhi specific antigens being investigated in any detail. Experiments utilizing convalescent serum from typhoid patients, indicates that individuals can respond to a range of *S*. Typhi antigens [26]. However, such responses appear to be variable and no obvious immuno-dominant antigens have been identified. Studies may be confounded by the fact that *in vitro *grown *S*. Typhi are used to measure responses, a factor that would eliminate the detection of any antigen exclusively expressed in the host. This could be an important consideration as many surface structures, e.g. pili, have such properties.

A potentially productive area may be to search for novel antigens which are specific for *S*. Typhi. Candidate targets could be identified initially by bioinformatics. Novel candidates could be expressed in systems such as yeast to minimize contamination with cross-reactive antigens. A pool of highly purified specific antigens could be screened using serum from typhoid patients and appropriate controls. Protein microarrays could be exploited in the screening [42]. Testing in a cohort of patients could reveal specific patterns or quantities of antibodies which would be indicative of typhoid infection. Ultimately, novel antigen(s) could be placed onto membrane to form the basis of a low cost rapid test. This is an open and uninvestigated area and with a suitable assay and patient material, it may be one of the most straightforward ways to initially develop a low cost and highly specific test.

## The limits of DNA detection

The detection of specific DNA sequences within the genome of *S*. Typhi would appear to be an attractive proposition. Is a robust DNA-based test a real option for routine typhoid diagnostics? Many *S*. Typhi PCR-based assays have targeted the *fliC *gene, utilizing nested primers to improve sensitivity [43-47]. There is an additional sensitivity benefit of PCR, in that it can theoretically amplify DNA from dead or unculturable bacteria. Various PCR-based studies on typhoid suggest that the assay is specific and sensitive and relatively straight forward to perform. Indeed, such studies have yielded sensitivities >90%. However, we believe PCR offers only limited potential for typhoid diagnostics. Currently there is no validated PCR test in common use, only in-house systems which are open to differing interpretation and none would meet the rigors of quality control to make this assay used worldwide.

Massi *et al*. utilized a real-time system based on *fliC *to detect *S*. Typhi in patients with clinically diagnosed typhoid [48]. They were able to amplify *fliC *from all culture-positive and negative blood samples tested but reported a higher gene copy number in culture positives (1,000 - 45,000), compared to negatives (<1000). However, this real-time PCR data is somewhat contradictory with the microbiological data, which demonstrates that bacteria/ml of blood is generally low with the majority of patients having <1 organism/ml of blood [21]. It is somewhat surprising that typhoid patients may have between a 1,000 to 45,000 times more dead bacteria than live bacteria in the blood.

We recently found disappointingly poor PCR sensitivity using a three color real-time PCR assay that was capable of detecting *S*. Typhi, *S*. Paratyphi A and an incorporated internal control [49]. When tested on spiked and control samples the assay demonstrated high specificity and sensitivity. However, when tested on DNA extracted from 2 ml of blood taken from 100 culture confirmed typhoid patients the sensitivity rate was less that 50%. Thus confirming that PCR results are related to the actual colony forming units found in the blood. The assay did, however, demonstrate 100% sensitivity on culture positive bone marrow samples, which are known to harbor significantly more bacteria [22]. For these reasons we believe that DNA amplification may not be an easy route towards developing a robust diagnostic. Collecting and then extracting DNA from a large volume of blood is not a straightforward option, due to large concentrations of human DNA. Analyzing stool or urine samples may be an alternative approach. A DNA or bacterial capture system or even a culture enrichment step prior to amplification may improve molecular sensitivity. However, molecular diagnostics are not a cost effective or a straightforward to perform as other methods, not every diagnostic laboratory in an endemic setting would be able to perform such an assay. However, if simplified and new technology is applied it an area that warrants further independent studies.

## Host factors other than antibodies

Is it possible to identify host specific responses to typhoid that are distinct from other febrile diseases such as malaria or dengue? If so, what sort of responses should we look for? Typhoid patients display a number of symptoms including fever and mount a number of immune and physiological responses. Such responses can be examined by simple stimulation assays, exploiting whole blood, cell fractions or serum. Currently, there has been no precise correlate of infection or biomarker for typhoid identified. An expansive, yet costly option would be to take an approach based on human microarrays [50]. Transcriptional analysis of RNA extracted from the blood of typhoid patients could be performed to identify specific genes, pathways, interactions or transcriptional regulatory hubs that are activated in the host during infection. Microarray data is often publicly available and comparative analysis with the transcriptional profile from patients with other diseases could be studied at databases such as InnateDB http://www.innatedb.ca/. Such analysis may highlight suitable targets that could be tracked in patients [51].

Mass spectrometry, proteomics or similar expression monitoring technologies could be applied to identify particular genes or pathways that are functionally activated during typhoid. Once a gene or transcriptional pathway is identified, expression could be monitored using DNA or protein probes. This approach may be a long term aim and comparative analysis with similar materials from other diseases would be an essential requirement. Blood would most likely be the assay material of choice and this in itself may present limitations if responses are localized to deeper tissues. However, this approach is highly novel, powerful and worthy of further investigation and investment.

An further alternative approach would be to identify potential biomarkers, i.e. discover a physiological signature or metabolic product associated with typhoid. The signature could be of host or bacterial origin or a combination of both that is/are produced in real time during infection. The science of host metabolomics is growing with the development of applications such as NMR and Mass Spectrometric technologies. Metabolomics could, theoretically, work on a range of bodily fluids, including blood and urine and may detect specific small or complex macromolecules. Some research groups have developed systems for identifying biomarkers in biological material from patients infected with various pathogens such as Tuberculosis [52,53]. Surface-enhanced laser desorption/ionization time-of-flight (SELDI-TOF) mass spectroscopy has also been utilized in studying SARS protein biomarkers, as reviewed by Mazzulli *et al*. [54]. SELDI-TOF may add insight into those proteins that are expressed in serum, blood, saliva, urine or any other biological material that may harbor specific markers for typhoid infections. Some early studies on pathogenesis and diagnostics did focus on the detection of *S*. Typhi antigens such as Vi in the urine of patients and this is worth revisiting in view of a substantial increase in the sensitivity of detection technologies [55,56].

## Typhoid carriers

What about the diagnosis of typhoid in carriers infected with *S*. Typhi or *S*. Paratyphi A [57,58]? Clearly, such individuals warrant special consideration as they are a silent threat to others in the population. Monitoring *S*. Typhi in the stool is one option but shedding may be low level or sporadic. Further, stool sampling at a routine level is expensive, time consuming and unpopular, although improved bacterial recovery methods could be one approach. We know of no obvious signature that can be used to categorically identify *S*. Typhi carriers. However, important studies have indicated that typhoid carriers may produce higher levels of Vi antibodies over extended periods compared to acutely infected patients [59,60]. This may be in part because Vi is a polysaccharide and the immune response to Vi is T cell-independent, stimulating poor memory. However, carriers may receive continual, natural boosting when the organisms are reseeded, potentially in high numbers (Figure 2), back into the intestinal tract. If we could develop simple, cheap and none invasive Vi antibody assays these may prove valuable in identifying carriers.

## Concluding remarks

The ultimate question is which direction do we follow in terms of developing typhoid diagnostics and how can these be applied to location with limited resources? In the short term, it appears that whilst current techniques are limited there is no real alternative without extensive research and culturing remains the inadequate gold standard. However, laboratories in developing countries with typhoid should be prepared to evaluate new diagnostics as they evolve. As a way forward for culture, it may be prudent to investigate specialized growth media that would favor the regeneration of *S*. Typhi from blood. Simple methods for enriching the small population of bacteria present in blood using simple direct enrichment procedures that do not rely on growth could be considered.

DNA methodology has specific limitations that are similar to those presented with bacterial culture. Advancement in this field would require the capture and amplification from a smaller number (maybe even a single organism) from blood or other bodily fluids. Such a task is not insurmountable but it will be a challenge to make it cost effective.

Serological advancements will rely on the identification of novel *S*. Typhi-specific antigens that are conserved and highly immunogenic in the human host. We will need simple methods to prepare highly purified antigens free of potentially cross reacting materials and antigen pools may be needed to increase sensitivity. Serological approaches may be more tractable to convert into a simple, cheap and rapid test. Host response assays will have to be developed through the application of genomics and highly sensitive Mass Spectrometric, NMR or similar sensitive physical assays. Looking for host or pathogen material in biological samples is an area that clearly warrants further investigation.

Once targets have been identified, the next limiting step, with respect to locations with limited resources is developing a reliable test that is affordable. With the identification of novel targets is should be feasible to create simple point of care assays aimed at these specific targets. However, making such tests that can be manufactured at a reasonable cost that can aid typhoid diagnostics in the locations where they are required most may add an additional hurdle.

## Competing interests

The authors declare that they have no competing interests.

## Authors' contributions

SB, MF and GD were responsible for the concept, the content and the writing of the manuscript. All authors have read and approved this manuscript.

## Pre-publication history

The pre-publication history for this paper can be accessed here:

http://www.biomedcentral.com/1471-2334/10/45/prepub
